# Sugar- and Intense-Sweetened Drinks in Australia: A Systematic Review on Cardiometabolic Risk

**DOI:** 10.3390/nu9101075

**Published:** 2017-09-28

**Authors:** Erin Hoare, Pia Varsamis, Neville Owen, David W. Dunstan, Garry L. Jennings, Bronwyn A. Kingwell

**Affiliations:** 1 Baker Heart and Diabetes Institute, Melbourne 3000, Australia; Pia.Varsamis@baker.edu.au (P.V.); Neville.Owen@baker.edu.au (N.O.); David.Dunstan@baker.edu.au (D.W.D.); Garry.Jennings@baker.edu.au (G.L.J.); Bronwyn.Kingwell@baker.edu.au (B.A.K.); 2 Department of Physiology, Monash University, Clayton 3800, Australia; 3 School of Medicine, Monash University, Clayton 3800, Australia; 4 Centre for Urban Transitions, Swinburne University, Hawthorn 3122, Australia; 5 Department of Epidemiology and Preventive Medicine, Monash University, Melbourne 3004, Australia; 6 Mary MacKillop Institute for Health Research, Australian Catholic University, Melbourne 3000, Australia; 7 Sydney Medical School, University of Sydney, Camperdown 2006, Australia

**Keywords:** sugar-sweetened beverages, intense-sweetened beverages, glycaemic control, cardiometabolic risk factors, systematic review

## Abstract

Sugar-sweetened beverages (SSBs) are consumed globally, and have been associated with adverse health outcomes, including weight gain, high blood pressure, type 2 diabetes (T2D), and cardiovascular disease (CVD). There is global variation in beverage formulation in terms of glucose and fructose concentration, which may pose unique health risks linked to glycemic control for Australian consumers. However, previous systematic reviews have overlooked Australian-based literature. A systematic review was performed to synthesise evidence for the associations between consumption of SSBs and intense-sweetened beverages with clinical cardiometabolic risk factors in the Australian population. Articles were sourced from Global Health, Health Source: Nursing/Academic Edition, Medline, and Culmative Index to Nursing and Allied Health Literature. To be eligible for review, studies had to report on the consumption of sugar-sweetened (including fruit juice and fruit drinks) and/or intense-sweetened beverages, and at least one clinical cardiometabolic risk factor. Eighteen studies were included in this review. Research has mostly focused on the relationship between SSB consumption and adiposity-related outcomes. No studies have examined indices of glycaemic control (glucose/insulin), and the evidence for the health impact of intense-sweetened drinks is limited. In addition, studies have primarily been of cross-sectional design, and have examined children and adolescents, as opposed to adult populations. In the Australian population, there is modest but consistent evidence that SSB consumption has adverse associations with weight, but there is insufficient data to assess relationships with cardiometabolic outcomes.

## 1. Introduction

Sugar-sweetened beverage (SSB) consumption is of major global public health interest, with research suggesting associations with adverse health outcomes including overweight/obesity, high blood pressure, type 2 diabetes, and cardiovascular disease [1,2,3]. SSBs include soft drinks, cordials, fruit juices and drinks (juice plus added sugar), sports drinks, and energy drinks, and are typically sugar-sweetened drinks, high in energy density, offering little to no nutritional value. Intense-sweetened drinks are sweetened with food additives that do not significantly contribute to the energy content of the drink [4], and as such are a popular alternative to SSBs, although their health impact at a population level is largely unknown. There is also evidence to suggest a decline in availability and intake of added sugars in Australia, including those deriving from SSBs [5], and evidence on health outcomes is needed to inform current and future policy debate.

There has been extensive synthesis of evidence for the relationship between SSBs and overweight/obesity [2,6,7]. Furthermore, systematic reviews have investigated the prospective relationship of SSB consumption with type 2 diabetes (T2D) [8] and metabolic syndrome [9], although evidence is inconsistent as to whether relationships are attenuated when indices of weight are included as a covariate [10,11]. Limitations of the literature to date are that SSB intake is often self-reported, and incomplete adjustments for lifestyle factors, including overall diet quality and physical activity that typically cluster with SSB consumption, are not always included. Additionally, rarely have systematic reviews included Australian-based studies, with evidence largely limited to Western Europe and North America [12]. There has been a recent growth in the number of Australian-based studies examining SSB consumption (for example, [13,14,15]) and associated health impacts. This, in addition to recent interest in a national obesity strategy including a sugar tax [16], make a systematic review both feasible and timely.

Beyond the impact of SSBs on energy balance and risk for obesity, the large blood glucose and insulin excursions that can result from SSB consumption may independently increase risk for the development of type 2 diabetes mellitus (T2D) and cardiovascular complications [17,18,19]. Australian formulations are predominantly sweetened with sugar-cane-derived sucrose, which is composed of one molecule of glucose and one molecule of fructose. High-fructose corn syrup is also made up of glucose and fructose, but contains a higher fructose-to-glucose ratio than sucrose. We have recently shown that Australian soft drinks have a 22% higher total glucose concentration compared to U.S.A. formulations, which are sweetened with high-fructose corn syrup [20]. Given glucose (but not fructose) rapidly elevates plasma insulin, Australian formulations may affect risk for metabolic diseases, including diabetes [18], in a manner that is distinct from high-fructose drinks, which promote lipid accumulation [21,22,23]. This is a distinct risk beyond the detrimental caloric impact of SSB consumption, and further rationalises a systematic review of Australian-based research.

A large proportion of the Australian population are SSB consumers, with the 2011–2012 Australian Health Survey findings indicating that 34% of the participating sample consumed an SSB on the day prior to interview [24]. Australian studies have shown that higher SSB consumption often co-occurs with greater levels of socio-economic disadvantage, lower levels of parental education, and environmental and contextual circumstances [24,25,26]. While current Australian trends suggest that intake of SSBs may be stable or declining among children and adolescents, there has been increased consumption of sports, energy and fruit drinks with added sugars, and intense-sweetened drinks in young adults [12]. Unlike SSB consumption patterns, consumption of intense-sweetened drinks increases from childhood/adolescence to early-mid adulthood. Furthermore, adult women are greater consumers of intense-sweetened drinks compared to adult men [27]. There is increasing availability of drinks containing a mixture of sugars and intense-sweeteners, which may confound self-report data adding complexity to analyses examining health associations.

This systematic review examines observational evidence, in the Australian context, of associations between SSBs and intense-sweetened beverages with clinical cardiometabolic risk factors, including body mass index (BMI), weight status, waist circumference, type 2 diabetes, blood pressure, blood glucose, insulin and dyslipidaemia. Where possible, the impact of physical activity, diet and other lifestyle behaviours are examined.

## 2. Materials and Methods

The present systematic review was conducted according to Preferred Reporting Items for Systematic Reviews and Meta-Analyses (PRISMA) guidelines for reporting systematic reviews [28]. Articles were sourced from four databases; Global Health, Health Source: Nursing/Academic Edition, Medline Complete, Cumulative Index to Nursing and Allied Health Literature (CINAHL). The search was limited to peer-reviewed articles published in the English language published since 1990 and before August 2017.

### 2.1. Eligibility Criteria

Articles were eligible for review if they reported original research, were published in peer-reviewed journals, and reported on Australian study samples. Studies had to report on consumption of SSBs (including soft drinks, fruit drinks, cordials and sports drinks), intense-sweetened beverages, and/or fruit juice, to be eligible for review. Studies were also required to report on at least one clinical cardiovascular or metabolic risk factor, including body mass index (BMI), weight status, waist circumference, type 2 diabetes, blood pressure, blood glucose, insulin, cholesterol (total cholesterol, low-density lipoprotein or high-density lipoprotein) and triglycerides. All observational studies that had potential to demonstrate evidence for relationships were identified. This included prospective and retrospective cohort studies, and longitudinal and cross-sectional studies. Clinical trials were ineligible for review due to our focus on population-level evidence in non-experimental settings. Qualitative studies were ineligible due to our focus on behavioural and clinical measures collected through quantitative methods.

Studies focusing only on specific groups, such as those experiencing a chronic disease, those in treatment programs, or those considered at high risk for cardiovascular disease without also including those at low or no risk, were ineligible for review. Given the known comorbidities and general poor health of those experiencing chronic disease, studies with such participants were considered outside the scope of this review. Studies that reported data across multiple countries were analysed, and if Australian-based data were possible to derive, such articles were included in the review.

### 2.2. Search Strategy and Study Selection

The search strategy is reported in Supplementary Figure S1. Following the PRIMSA guidelines, search terms were developed to match the eligibility criteria. A search of electronic databases was completed on August 2017 including databases previously listed. All databases were accessed through EBSCOHost. Search terms included types of beverage consumption, clinical cardiometabolic risk factors, and the Australian population. After removal of duplicates and initial title and abstract screening, two authors (Erin Hoare, Pia Varsamis) assessed subsequent full-text articles for inclusion in this review. Disagreements were resolved through a third reviewer (Bronwyn A. Kingwell). Reference lists of selected studies and previous systematic reviews were hand searched to identify additional studies eligible for review. The search terms used were as follows: “Overweight” OR “obes” OR “adiposity” OR “waist circumference” OR “waist-hip ratio” OR “body mass index” OR “BMI” OR “blood pressure” OR “hypertens” OR “glucose intolerance” OR “blood glucose” OR “insulin” OR “cholesterol” OR “lipoprotein” OR “triglycerides” OR “lipid metabolism” OR “cardio metabolic” OR “metabolic syndrome” OR “cardiovascular disease” OR “type 2 diabetes” OR “diabetes” OR “T2DM” OR “diabetes mellitus” AND “carbonated beverage” OR “sugar sweetened beverage” OR “soft drink” OR “sugar drink” OR “non-diet drink” OR “soda” OR “soda pop” OR “fizzy drink” OR “refreshment” OR “cola” OR “coke” OR “pepsi” OR “coca cola” OR “lemonade” OR “diet soft drink” OR “diet soda” OR “diet cola” OR “diet coke” OR “juice” OR “fruit juice” OR “fruit drink” OR “orange juice” OR “apple juice” OR “energy drink” OR “sport drink” OR “gatorade” OR “powerade” OR “red bull” OR “dietary sucrose” OR “sweetening agents” AND “Australia” OR “Oceania” OR “Indigenous” OR “Aboriginal” OR “Torres Strait Island”.

### 2.3. Data Extraction

A data extraction tool was used to extract the information from articles selected for review. The tool included information on (i) study characteristics (authors, year, and study aim/s); (ii) study design (including sample characteristics); (iii) measure of a clinical cardiovascular or metabolic risk factor or factors; (iv) measure of SSB and other beverage consumption; (v) included covariates; (vi) findings; and (vii) implications of findings. A narrative synthesis of findings is provided, according to the clinical cardiovascular or metabolic risk factor measured.

## 3. Results

**Figure 1:**
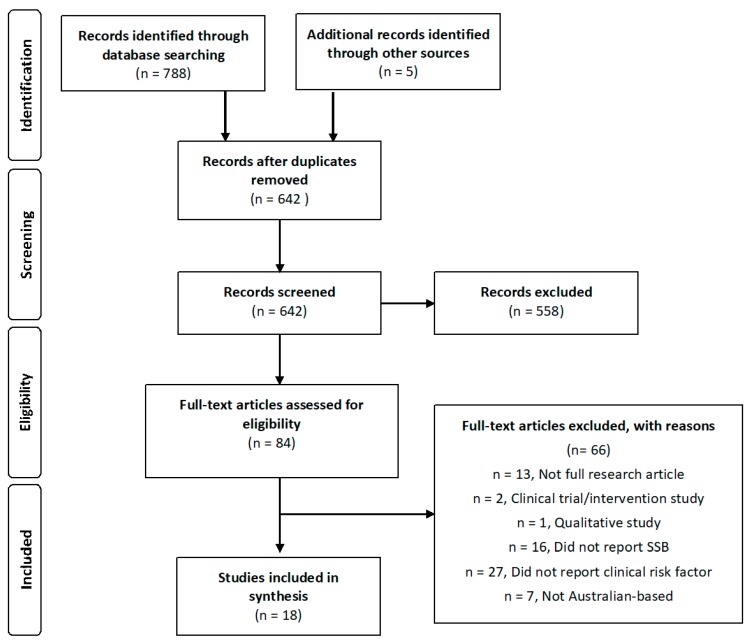
Preferred Reporting Items for Systematic Reviews and Meta-Analyses flowchart for article selection following database search.

The literature search retrieved 642 papers by database searching and other sources after duplicates were removed. Following title and abstract screening, 558 articles were excluded, leaving 84 articles for full-text screening. A further 66 records were excluded, for reasons including failing to report relevant beverage consumption or risk factor specifically (*n* = 27), not reporting specific SSB or other drink consumption (*n* = 16), not being full research articles (such as short reports, conference abstracts, letters to editor, and commentaries) (*n* = 13), examining populations other than Australia-based (*n* = 7), and two intervention/clinical trials and one qualitative study were excluded (Figure 1). No previous systematic reviews in relation to this research question were located. Excluded studies with reason for exclusion are reported in Supplementary Table S1.

### 3.1. Search Results

Eighteen articles were eligible and subsequently included in this review. Data extracted from the studies included can be found in Supplementary Table S2. Table 1 reports the outcomes measured in each of the respective studies. Thirteen cross-sectional and five longitudinal were included. Thirteen [14,15,29,30,31,32,33,34,35,36,37,38] examined children and adolescent populations aged between 4–18 years and five studies [13,39,40,41,42] examined adult populations. One [26] examined older adolescents and adults aged 16–65 years. All studies reported using population representative data, however three appeared to have investigated distinct research objectives but within the same dataset in Barwon South Region of Victoria [33,35,37] and two analysed the Western Australian Pregnancy Cohort (Raine) study data [15,39]. All studies included self-report measures of relevant beverage consumption, typically recorded as a frequency over a period of time. Drinks largely included all categories of interest (as described above in the Introduction) and studies that focused on particular drinks (e.g., fruit drinks, energy drinks, or intense-sweetened beverages only) are identified where relevant. Significance was assumed when *p* < 0.05.

### 3.2. Outcomes

#### 3.2.1. Adiposity-Related Risk Factors

Sixteen studies examined the relationship between SSBs and adiposity-related risk factors, including overweight/obesity [13,26,30,31,32,35,38], BMI or BMI z-score [14,15,33,36,37,39,41], waist circumference [15,42], and body fat percentage [36]. Only one study examined intense-sweetened drinks in addition to SSBs [26]. Anthropometric measures were collected by trained researchers in all studies but except three, which were self-reported [13,26,30]. These findings are summarised below for children/adolescents, and adult populations respectively.

Of the studies examining children and adolescents, 10 studies were found finding significant positive associations between SSB consumption and weight-related measures, including overweight/obesity defined by internationally recognised criteria (e.g., World Health Organization, International Obesity Taskforce) [30,31,32,35,38], higher BMI/BMI z-scores [14,15,29,36,37], greater waist circumference [15] and body fat percentage [36]. Clifton et al. (2011) [30] found that the proportion of SSB consumers (measured by 24 dietary recall) in the 6% of children who were obese was significant compared with the non-overweight children (59% vs. 47%, *p* < 0.05). Similarly, Grimes et al. (2013) [31] found that young people aged 2–16 years who had consumed more than 1 serving (>250 g) of SSBs during the previous day were 26% more likely to be overweight/obese (OR: 1.26, 95% CI: 1.03–1.53, *p* < 0.05). Hardy et al. (2012) reported a significant difference in high (five or more cups per day) levels of SSB intake (measured by food frequency questionnaire) between overweight (30%) and non-overweight (23%) young people (*p* < 0.05) [32]. Sanigorski identified an increased likelihood (OR: 2.2, 95%; CI: 1.3–3.9, *p* < 0.05) of overweight/obesity among children who had had more than 3 servings of SSB on previous day compared to non-consumers. Wheaton et al. (2015) demonstrated this relationship over time, with higher consumption patterns of SSBs being linked to overweight and obesity throughout early to middle childhood (Relative Risk Ratio: 1.13, SE: 0.06, *p* < 0.05) [38]. Importantly, these relationships were frequently shown to be significant after controlling for age, gender, and socio-economic status [31,35,38]. One study specifically examined Indigenous young people aged 3–6 years at baseline and 6–9 years at follow-up demonstrating less rapid rates of BMI increases among those children consuming less than 2 serves of SSBs per day, compared to children who consumed two or more glasses [29].

There was further evidence to suggest that, after controlling for total dietary intake/overall energy consumption, the relationships remained significant for overweight/obesity [31], body fat percentage [36], and BMI among adolescent girls [15]. A higher level of daily consumption of SSBs among children was associated with higher BMI z-scores, after controlling for maternal/paternal BMI scores (with each unit increase in SSB consumed, BMI z-score increased by 0.015 units, *p* < 0.01) [14]. One study showed a greater likelihood of overweight/obesity among those who had consumed more than one SSB on the previous day; however, this relationship was attenuated after controlling for physical activity [31]. Adolescent girls who moved into the highest tertile of daily average SSB consumption (>1.3 servings per day) from age of 14 to 17 years were at increased risk for overweight and obesity (OR: 4.8, 95% CI: 2.1–11.4, *p* < 0.05) [15].

Based on a single study, evidence on adults included findings that the overweight and obese were more likely to consume both sugar-sweetened and intense-sweetened (diet) soft drinks compared to their non-overweight/obese counterparts after controlling for gender, location, age group, and SES (OR: 1.7, 95% CI: 1.1–2.7, *p* < 0.05) [26]. One other study found that obese adults were more likely to consume SSBs than were normal-weight adults in South Australia (OR: 1.77, 95% CI: 1.56–2.02, *p* < 0.05) and Western Australia (OR: 1.53, 95% CI: 1.05–2.24, *p* < 0.05), but it was unclear what confounding variables were included in the analyses [13].

Higher SSB consumption was reported among individuals with higher BMI scores for men (b = 0.06, 95% CI = 0.04–0.08, *p* < 0.05) and women (b = 0.05, 95% CI = 0.05, 95% CI = 0.02–0.08) [41]; however, no difference in objectively measured BMI was found between energy drink consumers and non-consumers [39]. Moderate abdominal obesity among men (SSBs 5 or more times a week PR = 1.79, 95% CI: 0.90–3.56) and severe abdominal obesity among women (3–4 SSBs per week PR = 2.70, 95% CI: 1.96–3.71) were associated to higher levels of SSB consumption during TV after controlling for demographics [42].

#### 3.2.2. Blood Pressure

Two of the studies included in this review examined SSB consumption and blood pressure [15,40]. Ambrosini et al. (2013) reported a trend of higher systolic blood pressure among those in the higher SSB consumption categories at baseline when participants were aged 14 years (tertile 1, m = 112.3 mm Hg; tertile 2, m = 113 mm Hg; tertile 3, m = 113.8 mm Hg, *p*-value for trend *p* < 0.05), but without controlling for potential confounders [15]; diastolic blood pressure was similar across groups categorised by level of SSB consumption. Pase et al. (2015) examined fruit juice consumption and blood pressure among community-dwelling adults aged 50–70 years [40]. Those who consumed fruit juice daily, compared to those who consumed SSBs rarely or on occasions, had significantly higher central systolic blood pressure (*F*(2, 134) = 6.09, *p* < 0.01). Covariates including age, height, weight, mean arterial pressure, heart rate, treatment for lipids and treatment for hypertension were accounted for, and the findings remained significant; physical activity and overall dietary patterns were not included as covariates.

#### 3.2.3. Other Cardiovascular and Metabolic Risk Factors

Ambrosini et al. (2013) developed a composite measure of metabolic risk based on 2-step cluster analysis of BMI, systolic blood pressure, serum triglycerides, and other key risk factors (reported in Table 1), which was then used to classify participants into low and high metabolic risk clusters [15]. Adolescent females who moved into the top tertile of SSB consumption (>1.3 servings/day) between 14 to 17 years were at greater overall cardiometabolic risk (OR: 3.2, 95% CI: 1.6–6.2, *p* < 0.001). This finding was significant after controlling for age, pubertal status, physical fitness, dietary misreporting, maternal education, and family income. The relationship remained significant after further controlling for western dietary pattern, which was defined as high intake of fat, saturated fat, cholesterol, and refined sugars, and has been shown to correlate with SSB consumption; the relationship was non-significant among young men.

## 4. Discussion

Research on the health impacts of widely available Australian beverages has, to date, mostly focused on the relationship between SSB consumption and weight-based outcomes. No observational Australian-based studies have examined indices of glycaemic control (glucose/insulin), HDLc LDLc, and T2DM, and the evidence for the health impact of intense-sweetened drinks is limited. This is consistent with the international evidence, with weight-based outcomes having largely been the focus of international reviews [2,6], although evidence syntheses have emerged relating specifially to SSBs, T2DM [7,9], and metabolic risk [42]. Our review additionally found that studies have primarily been of cross-sectional design, and have examined children and adolescents, as opposed to adult populations. Overweight/obesity have been the predominant focus of these studies, which show that higher BMI is linked to increased SSB consumption, with some studies reporting significant associations after accounting for demographic factors, overall energy intake and maternal/paternal BMI. Studies have often, however, failed to include important covariates such as total energy intake, physical activity patterns, and other factors known to co-occur with SSB consumption. It is also likely that SSB consumption could simply be a marker of poor diet quality [43,44,45]. Without inclusion of total energy intake and controlling for known covariates, the interpretation of these specific associations requires caution. The evidence was limited by largely cross-sectional design, self-report measures, and heterogeneity in outcome measures, and was therefore considered low quality.

Additionally, higher blood pressure was associated with increased consumption of SSBs [15] and fruit juice [40], although these findings were limited to just two studies. Higher metabolic risk was associated with increased levels of SSB consumption among adolescent girls, but this was not found among adolescent boys, and was again limited to a single study [15]. Overall, however, there is only a modest body of findings on associations of consumption with cardiometabolic risk factors other than those related to weight. Such risk factors may not manifest until later in life [46], and longitudinal studies are needed to explore the relationships with SSB consumption. A lack of studies to date may reflect increased difficulty in capturing such information, which may require blood or urine collection. The feasibility of collecting anthropometric data compared to other cardiometabolic risk factors may explain this discrepancy in the evidence to date. This systematic review found limited Australian-based evidence on the metabolic consequences of higher SSB consumption, despite there being extensive international evidence [8,10,11]. This is an important finding, given consumption of Australian SSBs may cause greater elevation in glucose and insulin compared to U.S.A. formulations [47], and may therefore pose specific health risks. This lack of evidence is likely due to the limited number of studies available, as opposed to the non-existence of such relationships.

Physical activity levels can be important in mitigating the adverse effects of SSB consumption on obesity. For example, Grimes et al. reported that, among children and adolescents, the significant relationship of overweight/obesity with consuming more than one SSB on the previous day became non-significant after physical activity was included in analyses [31]. This highlights the need to examine effects of consumption of SSBs across the life span, given decreasing levels of physical activity with age, particularly for young women during adolescence [48]. Physical activity might mitigate the effects of SSBs during childhood, but if SSB consumption continues into adulthood, there may be adverse effects later in life when activity decreases. If, for example, declining activity levels are coupled with sustained SSB consumption a positive energy balance may lead to obesity and associated risk of complications.

To date, intense-sweetened beverages, fruit juice, and energy drinks have not been a primary focus of research, but given industry advertising and community perceptions that these are healthy options, such research is urgently required [24,40,49]. This is particularly the case given the trend of increased consumption of intense-sweetened beverages in Australian adults, especially among women [27]. Emerging international findings on the effects of intense-sweetened beverages are equivocal, showing both no health effects [50] and associations with elevated risk of glucose intolerance [51]. Further evidence from observational studies is clearly required, especially considering the findings of experimental studies suggesting detrimental metabolic consequences via mechanisms including modulation of the gut microbiome [51].

The associations between Australian SSB consumption and adiposity-related factors have not been well studied, representing a major evidence gap in light of international variations in SSB formulation and current national policy debate. The relationships between overweight/obesity and SSB consumption found in Australia are consistent with international evidence [2], and SSB consumption has also been linked to greater risk of metabolic syndromes [9] and T2D [8,10]; however, this is yet to be examined comprehensively from an Australian perspective. While the health effects of energy imbalance and overconsumption of added sugars have been well established, the findings of this review indicate that the health impacts of SSBs also need to be considered as one subset of a broader range of health behaviours. Evidence is emerging, for example, suggesting a relationship between SSBs and non-alcoholic fatty liver disease [52,53], signifying consumption patterns may co-occur with a broad range of clinical conditions, and that there is underlying complexity in behavioural risk and associated health outcomes. The evidence emerging from this review is timely in the context of international and national discussions regarding SSB taxation [54], global obesity prevention efforts [55], and projected trends in cardiovascular and metabolic diseases in Australia [56].

This systematic review sought to comprehensively analyse the Australian-based evidence between SSB consumption and clinical cardiovascular and metabolic risk factors. Our search strategy was designed to capture observational evidence providing insights into what might be the case for the broader Australian population, with inclusion criteria that had no limitations on study sample size, demographics, age and area of residence. As we have already highlighted, there is an increased need to examine Australian SSB consumption and associated health effects specifically, due to there being a uniquely Australian SSB formulation, and there being a lack of representation of Australian-based studies in systematic reviews and meta-analyses, to date. However, caution must be exercised in comparing these findings to what has been reported for studies conducted in other countries. All of the studies included involved participant self-report for SSB consumption, and there are known common problems with dietary recall [57]. A meta-analysis was not possible due to heterogeneity in outcomes measures and the cross-sectional nature of most of the findings examined precludes conclusions on causality. A quality of evidence grading was not feasible in this systematic review due to the specific focus on observational studies that were largely heterogonous in outcome measures.

## 5. Conclusions

The findings of this review indicate that the relationships of cardiovascular and metabolic risk with consumption are complex, and need to be examined in the context of broader health behaviours. While evidence suggests significant positive associations between SSB consumption and weight-related outcomes, particularly among children and adolescents, studies have often failed to control for known contributing factors such as total energy intake and physical activity. There is a gap in Australian-based research examining the specific associations of SSB consumption with cardiometabolic risk factors other than those that are weight-related. This is an important avenue for future research, given the significant, albeit limited, evidence that is available specifically on what the situation is in Australia. Furthermore, there is a dearth of relevant findings on the health impacts of intense-sweetened drink, sports and energy drink, and fruit juice consumption. This points to another important direction for future research, especially given the increasing population-level consumption of such beverages, and in light of their promotion by the drinks industry as healthy alternatives to SSBs. This evidence synthesis is timely with international and national debate regarding fiscal-policy actions targeting SSBs in the context of public health concerns and global obesity prevention efforts.

## Figures and Tables

**Table 1:** Clinical cardiometabolic risk factor measured and included covariates.

Study (Author/Year)	*n*	BMI/Overweight/Obesity		Waist Circumference	T2DM	Blood Pressure	Insulin Resistance	Glucose	Glycaemic Index	Triglycerides	Cholesterol	Metabolic Syndrome	Controlled for Physical Activity	Controlled for Diet	Other
		Obj	Sub												
Ambrosini et al. (2013) [15]	1433	√		√		√	√	√		√	√	√	√	√	√ ^1^
Cleland et al. (2008) [42]	2001			√											√ ^2^
Clifton et al. (2011) [30]	4400		√												
French et al. (2013) [26]	1015		√												
Gearon et al. (2013) [41]	30,630	√													√ ^3^
Grimes et al. (2013) [31]	4283	√											√	√	√ ^4^
Hardy et al. (2012) [32]	1568	√													
Jensen et al. (2013) [33]	1465	√													√ ^5^
Jones et al. (2016) [34]	1876								√						
Millar et al. (2014) [14]	4164	√													
Pase et al. (2015) [40]	160					√									√ ^6^
Pollard et al. (2016) [13]	13,596		√												
Sanigorski et al. (2007) [35]	1944	√													
Sluyter et al. (2013) [37]	1673	√													√ ^7^
Trapp et al. (2014) [39]	1234	√													
Thurber et al. (2017) [29]	887	√													
Wheaton et al. (2015) [38]	4169	√													√ ^8^
Zheng et al. (2015) [36]	158	√												√	√ ^9^

Obj, objectively measured; Sub, subjectively measured; ^1^ Adjusted for age, pubertal stage, dietary misreporting, maternal education, and family income. ^2^ Adjusted for age, education, occupation, and TV viewing time (h). ^3^ Adjusted for age and smoking status. ^4^ Adjusted for age, gender, education. ^5^ Adjusted for age, gender, SES. ^6^ Adjusted for age, gender, height, weight, mean arterial pressure, heart rate, treatment for lipids and hypertension. ^7^ Adjusted for SES and “being teased” but were removed when no changes were observed to analyses. ^8^ Adjusted for gender, age, ethnicity, parental BMI. ^9^ Adjusted for baseline age, gender, BMI z-score, SES, maternal age at birth, parental education level, presence of gestational diabetes, breastfeeding characteristics, pubertal status.

## Supplementary Materials

The following are available online at www.mdpi.com/2072-6643/9/10/1075/s1, Table S1: Excluded studies with reasons for exclusion. Table S2: Data extraction results for included studies. Figure S1: Example search strategy.
