# Supplementary material for: Sugar- and Intense-Sweetened Drinks in Australia: A Systematic Review on Cardiometabolic Risk
Source: Nutrients. 2017 Sep 28;9(10):1075. doi: 10.3390/nu9101075 (PMC5691692; doi:10.3390/nu9101075)
Supplement: Supplementary file 1 [file nutrients-09-01075-s001.zip › nutrients-220956-supplementary.pdf]

Supplementary file 1.

Excluded studies with reasons

	**Citation**	**Reason for exclusion**
1	(2004). "Tomato Juice for Diabetics." Saturday Evening Post **276**(6): 24-24.	Not full original research report.
2	(2012). "Soft drinks increase risk of heart disease." Australian Nursing Journal **19**(10): 44-44.	Not full original research report.
3	(2015). "One less sugary drink a day cuts risk of diabetes." Australian Nursing & Midwifery Journal **22**(11): 34-34.	Not full original research report.
4	Allman-Farinelli, M., et al. (2016). "Weight-Related Dietary Behaviors in Young Adults." Current Obesity Reports **5**(1): 23-29.	Narrative review.
5	Baghurst, K. I. and S. J. Record (1983). "Intake and sources, in selected Australian subpopulations, of dietary constitutents implicated in the etiology of chronic diseases." Journal of Food and Nutrition **40**(1): 1-15.	Did not include CVD risk factor.
6	Baur, L. A. (2005). "Predictors of weight gain in childhood and adolescence." Asia Pacific Journal Of Clinical Nutrition **14**: S33-S33.	Conference abstract.
7	Bell, L. K., et al. (2013). "Dietary patterns of Australian children aged 14 and 24 months, and associations with socio-demographic factors and adiposity." European Journal Of Clinical Nutrition **67**(6): 638-645.	Did not examine SSB consumption uniquely nor by CVD risk factor.
8	Bolton, K. A., et al. (2016). "The association between self-reported diet quality and health-related quality of life in rural and urban Australian adolescents." The Australian Journal Of Rural Health.	Did not examine CVD risk factor.
9	Cacavas, K., et al. (2011). "Tongan adolescents' eating patterns: opportunities for intervention." Asia-Pacific Journal of Public Health **23**(1): 24-33.	Not Australian-based.
10	Cameron, A. J., et al. (2012). "Socioeconomic variation in diet and activity-related behaviours of Australian children and adolescents aged 2-16 years." Pediatric Obesity **7**(4): 329-342.	Did not examine CVD risk factor
11	Cameron, A. J., et al. (2011). "Clustering of obesity-related risk behaviors in children and their mothers." Annals of Epidemiology **21**(2): 95-102.	Did not uniquely examine SSB consumption.
12	Campbell, K. J., et al. (2006). "Family food environment and dietary behaviors likely to promote fatness in 5-6 year-old	Did not examine CVD risk factor.

	children." International Journal Of Obesity (2005) **30**(8): 1272-1280.	
13	Chan, L., et al. (2011). "Maternal Feeding Practices and Feeding Behaviors of Australian Children Aged 12-36 Months." Maternal & Child Health Journal **15**(8): 1363-1371.	Did not examine CVD risk factor.
14	Cobiac, L., et al. (2003). "Sugars in the Australian diet: results from the 1995 National Nutrition Survey." Nutrition & Dietetics **60**(3): 152-173.	Did not report SSB consumption specifically.
15	Collins, K., et al. (2014). "Food and beverage portion sizes in Australian children: a secondary analysis of 1995 and 2007 national data." BMC Public Health **14**: 517-517.	Did not include CVD risk factor.
17	Denney-Wilson, E., et al. (2009). "Influences on consumption of soft drinks and fast foods in adolescents." Asia Pacific Journal Of Clinical Nutrition **18**(3): 447-452.	Did not include CVD risk factor.
18	Fayet, F., et al. (2013). "Australian children who drink milk (plain or flavored) have higher milk and micronutrient intakes but similar body mass index to those who do not drink milk." Nutrition Research **33**(2): 95-102.	No SSB.
19	Flood, V. M., et al. (2010). "Food and nutrient consumption trends in older Australians: a 10-year cohort study." European Journal Of Clinical Nutrition **64**(6): 603-613.	Did not report SSB consumption specifically (nor CVD risk).
20	Fonda, A. R., et al. (2010). Using child health checks to assess the prevalence of overweight and obesity among urban Indigenous children. Medical Journal of Australia. Pyrmont; Australia, Australasian Medical Publishing Co. **192:** 596-596	Letter to editor.
21	Gopinath, B., et al. (2013). "Carbohydrate nutrition and development of adiposity during adolescence." Obesity **21**(9): 1884-1890.	Did not report SSB consumption specifically.
22	Gopinath, B., et al. (Niven, P., et al. (2015). "What factors are associated with frequent unhealthy snack-food consumption among Australian secondary-school students?" Public Health Nutrition **18**(12): 2153-2160.2014). "Influence of obesogenic behaviors on health-related quality of life in adolescents." Asia Pacific Journal Of Clinical Nutrition **23**(1): 121-127.	Did not include CVD risk factor.
23	Gopinath, B., et al. (2014). "Parental history of hypertension and dietary intakes in early adolescent offspring: a population-based study." Journal Of Human Hypertension **28**(12): 721-725.	Measured parental hypertension and child’s dietary behaviours.

24	Hardy, L. L., et al. (2014). "Temporal trends in weight and current weight-related behaviour of Australian Aboriginal school-aged children." The Medical Journal Of Australia **200**(11): 667-671.	Did not include CVD risk factor association with SSB consumption.
25.	Hebden, L., et al. (2013). "A fizzy environment: availability and consumption of sugar-sweetened beverages among school students." Preventive Medicine **56**(6): 416-418.	Did not examine CVD risk factor.
26.	Hebden, L., et al. (2013). "Validity and reproducibility of a food frequency questionnaire as a measure of recent dietary intake in young adults." PLoS ONE **8**(9): e75156-e75156.	Validity/reliability study.
27.	Hodge, A., et al. (2011). "Plasma carotenoids are associated with socioeconomic status in an urban Indigenous population: an observational study." BMC Public Health **11**: 76-76.	Did not report SSB.
28.	Hodge, A. M., et al. (2004). "Glycemic index and dietary fiber and the risk of type 2 diabetes." Diabetes Care **27**(11): 2701-2706	Did not report SSB consumption.
29	Hosking, D. and V. Danthiir (2013). "Retrospective lifetime dietary patterns are associated with demographic and cardiovascular health variables in an older community-dwelling Australian population." The British Journal Of Nutrition **110**(11): 2069-2083.	Did not report SSB specifically.
30.	Innes-Hughes, C., et al. (2011). "Children's consumption of energy-dense nutrient-poor foods, fruit and vegetables: are they related? An analysis of data from a cross sectional survey." Health Promotion Journal of Australia **22**(3): 210-216.	Did not report CVD risk factor.
31.	Jensen, B. W., et al. (2012). "Consumption patterns of sweet drinks in a population of Australian children and adolescents (2003-2008)." BMC Public Health **12**(771): (12 Se-(12 Se.	Did not report CVD risk factor.
32.	Katzmarzyk, P. T., et al. (2016). "Relationship between Soft Drink Consumption and Obesity in 9-11 Years Old Children in a Multi-National Study." Nutrients **8**(12).	Australian-data not possible to extract.
33.	Kent, L. M. and A. Worsley (2009). "Trends in BMI, diet and lifestyle between 1976 and 2005 in North Sydney." Asia Pacific Journal Of Clinical Nutrition **18**(3): 453-461.	Did not report SSB specifically.
34.	Kourouniotis, S., et al. (2016). "The importance of taste on dietary choice, behaviour and intake in a group of young adults." Appetite **103**: 1-7.	Did not include CVD risk factor.
35.	Landon, S. (2007). Fruit juice nutrition and health. Food Australia. Alexandria;	Review

	Australia, Australian Institute of Food Science and Technology Inc. **59:** 533-538.	
36.	Lei, L., et al. (2016). "Dietary intake and food sources of added sugar in the Australian population." The British Journal Of Nutrition **115**(5): 868-877.	Did not include CVD risk factor.
37.	Louie, J. C. Y., et al. (2008). "Delayed effects of coffee, tea and sucrose on postprandial glycemia in lean, young, healthy adults." Asia Pacific Journal Of Clinical Nutrition **17**(4): 657-662.	Clinical trial.
38.	Louie, J. C. Y., et al. (2015). "Intake and sources of added sugars among Australian children and adolescents." European Journal Of Nutrition.	Did not include CVD risk factor.
39.	Louie, J. C. Y. and L. C. Tapsell (2015). "Intake of total and added sugars and nutrient dilution in Australian children and adolescents." The British Journal Of Nutrition **114**(11): 1875-1886.	Did not report SSB consumption specifically.
40.	McNaughton, S. A., et al. (2008). "Dietary patterns, insulin resistance, and incidence of type 2 diabetes in the Whitehall II Study." Diabetes Care **31**(7): 1343-1348.	Not Australian-based.
41.	McNaughton, S. A., et al. (2009). "Food patterns associated with blood lipids are predictive of coronary heart disease: the Whitehall II study." British Journal of Nutrition **102**(4): 619-624.	Not Australian-based.
42.	McNeill, T. J. and W. S. Shrapnel (2015). "Apparent consumption of refined sugar in Australia (1938-2011)." European Journal Of Clinical Nutrition **69**(11): 1233-1237.	No CVD risk factor.
43	Nghiem, S. and R. Khanam (2016). "Childhood obesity and the income gradient: evidence from Australia." Applied Economics **48**(50): 4813-4822.	Did not report CVD risk factor.
44	Niven, P., et al. (2015). "What factors are associated with frequent unhealthy snack-food consumption among Australian secondary-school students?" Public Health Nutrition **18**(12): 2153-2160.	Did not report SSBs.
45.	Miura, K., et al. (2012). "Socio-economic differences in takeaway food consumption among adults." Public Health Nutrition **15**(2): 218-226.	No CVD risk factor.
46.	Myers, J., et al. (2014). "Early childhood nutrition concerns, resources and services for Aboriginal families in Victoria." Australian And New Zealand Journal Of Public Health **38**(4): 370-376.	No associations between CVD risk factor and SSB (qualitative study).
47.	Novotny, R. and C. E. S. Oshiro (2009). Fruit intake for healthy body size in the Pacific region. Proceedings of the tropical fruits in human nutrition and health conference 2008, Couran Cove	Not original research study. (Conference)

	Island Resort, Gold Coast, Australia, 8-11 November 2008. Brisbane; Australia, The State of Queensland, Department of Employment, Economic Development and Innovation: 23-29.	
48.	O'Leary, F., et al. (2012). "Sugary drink consumption behaviours among young adults at university." Nutrition & Dietetics **69**(2): 119-123.	Did not include CVD risk factor.
49.	Panagiotopoulos, C., et al. (2014). "Cardiovascular risk factors and health behaviours in elementary school-age Inuvialuit and Gwich'in children." Paediatrics & Child Health **19**(5): 256-260.	Not Australia based.
50.	Pearson, N., et al. (2011). "Tracking of children's body-mass index, television viewing and dietary intake over five-years." Preventive Medicine **53**(4/5): 268-270.	Did not examine association with CVD risk factor.
51.	Pengpid, S. and K. Peltzer (2015). "Overweight and Obesity and Associated Factors among School-Aged Adolescents in Six Pacific Island Countries in Oceania." International Journal Of Environmental Research And Public Health **12**(11): 14505-14518.	Not Australian-based.
52.	Pettigrew, S., et al. (2015). "Factors influencing the frequency of children's consumption of soft drinks." Appetite **91**: 393-398.	Did not examine CVD risk (only parent's BMI).
53.	Poppitt, S. D. (2015). "Beverage consumption: are alcoholic and sugary drinks tipping the balance towards overweight and obesity?" Nutrients **7**(8): 6700-6718.	Narrative review.
54.	Rangan, A. M., et al. (2008). "Consumption of 'extra' foods by Australian children: types, quantities and contribution to energy and nutrient intakes." European Journal Of Clinical Nutrition **62**(3): 356-364.	Not CVD risk factor association.
55.	Renuka, V., et al. (2004). "Blood pressure responses in healthy older people to 50 g carbohydrate drinks with differing glycaemic effects." British Journal of Nutrition **92**(2): 335-340.	Trial.
56.	Ridoutt, B., et al. (2016). "Changes in food intake in Australia: comparing the 1995 and 2011 national nutrition survey results disaggregated into basic foods." Foods **5**(2): 40-40.	No CVD risk factor association.
57.	Rissel, C. E., et al. (2012). "Soft drink consumption and obesity in NSW school students." The Medical Journal Of Australia **196**(3): 171-172.	Letter to the editor.
58.	Salmon, J., et al. (2006). "Television viewing habits associated with obesity risk factors: a survey of Melbourne	Did not report association with CVD risk factor.

	schoolchildren." Medical Journal of Australia **184**(2): 64-67.	
59.	Silva-Sanigorski, A. M. d., et al. (2011). "Splash!: a prospective birth cohort study of the impact of environmental, social and family-level influences on child oral health and obesity related risk factors and outcomes." BMC Public Health **11**(505): (27 June 2011)-(2027 June 2011).	Methodology paper.
60.	Spurrier, N. J., et al. (2008). "Relationships between the home environment and physical activity and dietary patterns of preschool children: a cross-sectional study." International Journal of Behavioral Nutrition and Physical Activity **5**(31): (30 May 2008)-(2030 May 2008).	No CVD risk factor.
61.	Tam, C. S., et al. (2006). "Soft drink consumption and excess weight gain in Australian school students: results from the Nepean study." International Journal Of Obesity (2005) **30**(7): 1091-1093.	Not a full research article.
62.	Taylor, A. W., et al. (2014). "Ten-year trends in major lifestyle risk factors using an ongoing population surveillance system in Australia." Population Health Metrics **12**: 1-21.	Did not report association between SSB and CVD risk factor.
63.	Trapp, G. S. A., et al. (2014). "Energy drink consumption is associated with anxiety in Australian young adult males." Depression And Anxiety **31**(5): 420-428.	Did not report CVD risk factor. (Mental health)
64.	Vella, S. A., et al. (2013). "Associations between sports participation, adiposity and obesity-related health behaviors in Australian adolescents." International Journal of Behavioral Nutrition and Physical Activity **10**(113): (2 October 2013)-(2012 October 2013).	Did not report CVD risk factor.
65.	Woolley, S. E., et al. (2013). "Fructose intake and food sources in West Australian adolescents." Nutrition & Dietetics **70**(2): 139-145.	Did not report SSB specifically (total fructose intake)
66.	Zheng, M., et al. (2015). "Substituting sugar-sweetened beverages with water or milk is inversely associated with body fatness development from childhood to adolescence." Nutrition (Burbank, Los Angeles County, Calif.) **31**(1): 38-44.	Not Australian-based.

Supplementary File 2 – Results Table

Author, year, aim(s)	Methods	SSB Measure	CVD risk measure	Findings	Implications
Ambrosini, G. L., et al. (2013)<br><br>Aim: To test the hypothesis that higher SSB intakes are associated with increases in cardiometabolic risk factors between 14 and 17 y of age	*Design:* longitudinal<br><br>*Data source:* Adolescent offspring from the Western Australian Pregnancy Cohort study when adolescents were 14 years (2003-2006) and 17 years (2006-2009).<br><br>*Sample size:* 1433<br><br>*Age:* 14-17 years<br><br>*Gender:* 48% female	Food frequency questionnaire asked frequency of consumption and usual portion size of all carbonated drinks, cordials or squash and fruit juice drinks (excluding 100% juice). Items were combined to estimate the daily intake of SSBs, (g/d).	Study participants attended the study clinic for physical assessments at both follow-ups. Calibrated measurements of height and weight were made by using electronic chair scales and a stadiometer. *Overweight/obesity* was defined using International Obesity Task Force cut-offs for BMI. *Waist circumference* was measured. *Blood pressure* was measured using an oscillometric spygmanometer after subject's rested supine for 5 mins. Blood samples were collected at both follow-ups after an overnight fast. *Serum insulin, glucose, triglycerides, HDL cholesterol*, and calculated *LDL cholesterol* were conducted.	The average SSB intake in consumers (89%) was 335 g/d or 1.3 servings/d. Girls who moved into the top tertile of SSB consumption (>1.3 servings/d) between 14 and 17 y of age had increases in BMI (3.8%; 95% CI: 1.8%, 5.7%), increased overweight and obesity risk (OR: 4.8, 95% CI: 2.1, 11.4), and greater overall cardiometabolic risk (OR: 3.2; 95% CI: 1.6, 6.2) (all P-trend ≤ 0.001). Girls and boys who moved into the top tertile of SSB intake showed increases in triglycerides (7.0-8.4%; P-trend ≤ 0.03), and boys showed reductions in HDL cholesterol (-3.1%; 95% CI: -6.2%, 0.1%; P-trend < 0.04) independent of BMI. Some associations were attenuated after adjustment for major dietary patterns.	Increased SSB intake may be an important predictor of cardiometabolic risk in young people, independent of weight status.
Cleland, V. J., et al. (2008)<br><br>Aim: To determine whether the association between TV viewing and abdominal obesity in young adults is mediated by food and beverage consumption during TV viewing time or by a reduction in overall leisure-time physical activity (LTPA).	*Design:* cross-sectional<br><br>*Data source:* Data collected from young Australian adults from 2004 to 2006 as part of the Childhood Determinants of Adult Health study.<br><br>*Sample size:* 2001	Participants reported how often they consumed a soft drink during TV viewing (every day, 5-6 times per week, 3-4 times per week, 1-2 times per week or never).	Waist circumference was measured by trained researchers. Abdominal obesity was classified with WHO cut-offs.	Increased soft drink consumption during TV viewing time was significantly associated to moderate abdominal obesity among men (soft drink 5 or more times a week PR=1.79 (0.90-3.56), compared to zero times per week). Among women soft drink consumption during TV viewing time was significantly associated to	The association between TV viewing and WC in young adults may be partially explained by food and beverage consumption during TV viewing but was not explained by a reduction in overall LTPA. Other behaviours likely contribute to the association between TV viewing and obesity.

	Age: 26-36 y<br><br>*Gender:* 53% female			severe abdominal obesity (3-4 times per week PR=2.70 (1.96-3.71)).	Not necessarily TV viewing time as energy intake overall was not accounted for.
Clifton, P. M., et al. (2011)<br><br>Aim: To (1) examine SSB intakes in the 2007 Australian Children's Nutrition and Physical Activity Survey (2) relate SSB intake to rates of overweight and obesity, socio-economic status (SES), TV viewing time, and activity levels and (3) compare 2007 SSB intakes with data from the 1995 National Nutrition Survey.	*Design:* cross-sectional<br><br>*Data source:* 2007 Australian Children's Nutrition and Physical Activity Survey and 1995 National Nutrition Survey<br><br>*Sample size:* 4,400<br><br>*Age:* 2-16 y<br><br>*Gender:* not reported	Computer assisted 24 h dietary recall.	Self-reported height and weight, converted into BMI and weight status cut-offs were defined for Cole et al.	Activity levels were unrelated to SSB consumption. Television viewing was positively related to soft drink consumption with a difference of 55 g/day from bottom to top tertile of time spent TV viewing ($p = 0.015$) in children aged 9-16 years. 55% of SSB consumption occurred at home and 10% occurred at school. Lower SES status was associated with a greater prevalence of SSB consumption- 30% for the lowest SES quartile vs 19% in the highest quartile. The proportion of overweight who consumed SSBs (which excludes 100% fruit) was not different from the non-overweight children although the proportion of SSB consumers in the 6% of children who were obese was significant compared with the non-overweight children (59% vs 47%, $p < 0.05$).	This study provides little support to conclude that overweight in children is currently being driven by excessive SSB consumption although it may be factor in some obese children.
French, S., et al. (2013)<br><br>Aim: To examine soft drink consumption across age,	*Design:* Cross-sectional<br><br>*Data source:* Cross-sectional	Respondents were asked separately rate their consumption of SSBs and diet soft drinks. Consumption of	Height and weight were self-reported. BMI was calculated and underweight, acceptable weight and overweigh/obesity	On average, diet soft drinks were consumed on 4 days per week and SSBs were consumed on 2 days per week.	This study identified a high prevalence of soft drink consumption within the population (74.5%). These results

gender, socioeconomic, and body weight status groups within an adolescent and adult population.	telephone survey in Western Australia, participants selected through random dialling on two occasions (May, 2010 and February, 2011).<br><br>*Sample size:* 1,015<br>*Age:* 16-65 y<br><br>*Gender:* 50% female	non-carbonated beverages such as cordials, sweetened fruit and sports drinks, or flavoured water were not considered. Asked the number of days in the previous week in the previous week on which they has such drinks and the amount consumed.	were calculated using WHO criteria.	After adjustment for other factors (gender, age, location, SES), overweight or obese respondents were more likely than not (OR, 2; 95% CI, 1.2–3.3; P < .01) to consume soft drinks than those of acceptable weight. Overweight respondents were 1.7 times more likely (95% CI, 1.1–2.7; P ¼ .02) to consume a combination of diet and sugar-sweetened drink compared with those of acceptable weight.	identify young people (16–24 years of age), as well as males, as population groups to target through public health interventions to reduce soft drink consumption.
Gearon, E., et al. (2013)<br><br>Aim: This study aimed to ascertain the contribution of specific dietary elements and leisure-time physical activity (LTPA) to variations in obesity with education in the baseline (1990-1994) Melbourne Collaborative Cohort Study (MCCS).	*Design:* cross-sectional<br><br>*Data source:* Melbourne Collaborative Cohort Study<br><br>*Sample size:* 30,630<br><br>*Age:* 52-57y<br><br>*Gender:* 60.4% female	Data was collected by trained interviewers, and included a food frequency questionnaire.	Height and weight were objectively measured. BMI was used as a continuous measure.<br>Self-reported history of angina, stroke, heart attack, cancer and diabetes were also collected.	Association between soft drink consumption and BMI was significant for men (β=0.06, OR=0.04-0.08) and women (β=0.05, OR=0.02-0.08).<br>Association between diet soft drink consumption and BMI was significant for men (β=0.16, OR=0.13-0.18) and women (β=0.20, OR=0.18-0.23).	Both diet soft drinks and soft drinks consumption was associated with higher BMI. This study did not control for important confounders, but demonstrates the possibility that diet soft drinks may not be a healthy alternative.
Grimes, C. A., et al. (2013)<br><br>Aim: To determine the association among dietary salt, fluid, and sugar-sweetened beverage (SSB) consumption and weight status in a nationally representative sample of	*Design:* Cross-sectional<br><br>*Data source:* 2007 Australian National Children's Nutrition and Physical Activity Survey.	Consumption of dietary salt, fluid, and SSB was determined via two 24-hour dietary recalls.	Weight and height were measured objectively and BMI was used to determine weight status (International Obesity Task Force reference cut-offs).	Of the 4283 participants, 62% reported consuming SSBs. Older children and those of lower socioeconomic status (SES) were more likely to consume SSBs (both Ps < .001). Participants who consumed more than 1 serving (>250	SSB consumption explained greater variance among those overweight/obese compared to normal weight after controlling for various confounders, however this was non-significant after PA.

Australian children aged 2 to 16 years	*Sample size:* 4283<br><br>*Age:* 2-16 years<br><br>*Gender:* 48.3%			g) of SSB were 26% more likely to be overweight/obese (odds ratio: 1.26, 95% confidence interval: 1.03-1.53), adjusted for age, gender, SES, and energy derived from sources other than SSBs. The relationship was non-significant after further controlling for moderate intensity PA.	
Hardy, L. L., et al. (2012)<br><br>Aim: To determine the prevalence, sociodemographic distribution, and co-occurrence of five potentially obesogenic behavioural risk factors (low physical activity, high screen time, low fruit and vegetable intake, high soft drink consumption, and high snack intake) among adolescents.	*Design:* cross-sectional<br><br>*Data source:* Representative population-based survey of school students in NSW.<br><br>*Sample size:* 1568<br><br>*Age:* Grade 6, 8, 10<br><br>*Gender:* 51% female	Dietary data was collected using questions based on food frequency questionnaire. Students were asked to report the number of cups usually consumed and responses were categorised into five or more cups per week.	Objectively measured height and weight and BMI was categorised according to International Obesity Task Force weight status categories.	For males, non-significant difference in the proportion of overweight/obese compared to not overweight in the prevalence of high soft drink intake.<br>For females, there was a significant difference in high soft drink intake between overweight/obese (30%) and not overweight (23.1%) (p=0.027).	Differences in SSB consumption by weight status was significant for females, but not males.
Jensen, B. W., et al. (2013).<br><br>Aim: To examine whether baseline (T1) or 2-year change in sweet drink intake in children and adolescents was associated with age- and gender-standardized body mass index (BMIz) at time two (T2), 2 years later.	*Design:* Longitudinal<br><br>*Data source:* Comparison groups from the Be Active Eat Well and It's Your Move projects.<br><br>*Sample size:* 1465	Assessed by 'how many cans of soft drink did the child drink yesterday?' diet soft drinks were not included, and 'how many serves of fruit juice or cordial did the child drink yesterday?' And for IYM 'on the last school day how many glasses or cans of non-diet diet soft drinks did	Weight and height were objectively measured. Weight status was derived from WHO and IOTF.	No association was observed between T1 sweet drink intake and BMIz at T2 among children or adolescents. Children (4-11 years) from higher SES families who increased their sweet drink intake over the two year period recorded a trend towards a higher BMIz after two years, relative to those reporting no change in	This study showed limited evidence of an association between sweet drink intake and BMIz. However, the association is complex and may be confounded by both dietary and activity behaviours.

	Age: 4-18 years<br><br>*Gender:* 49.8% female, 53.4% female	you have?' and ' on the last school day how many glasses of fruit drinks of cordial did you have?'		take. Usual intake of juice/cordial at more than twice a week was association with increased BMIz 2 years later among children.	
Jones, M., et al. (2016)<br><br>Aim: To examine the dietary glycemic index (GI) and glycaemic load (GL) of Australian children and adolescents as well as the major food groups contributing to GL in the 2011-2012 Australian Health Survey	*Design:* cross-sectional<br><br>*Data source:* 2011-12 Australian Health Survey<br><br>*Sample size:* 1876<br><br>*Age:* 2-18 years<br><br>*Gender:* 49% female	Multiple-pass 24-h recall in a face-to-face interview.	GI of foods was assigned based on a step-wise published method using values from common GI databased.	As age increased, all varieties of fruit and vegetable juices and drinks contributed less and less to dietary GL. In contrast SSB contributed more and more as age increased. Children aged 4-8 years were found to have lower intake of sweetened beverages than their counterparts aged 9-18 years. Participants aged 14-18 years also had higher intake of sweetened beverages than 9-13 year olds. No significant gender differences were found.	This study was limited in that dietary assessment in children in difficult and parent reporting may not be reliable.<br>Results indicated that 3% of children and adolescents were meeting low GI eating pattern. Both GI and GL increased with age driven in part by SSB. On average, energy dense nutrient poor foods contributed almost a quarter of dietary GL.
Millar, L., et al. (2014)<br><br>Aim: To determine the relationship between frequency of consumption of sugar sweetened beverages (SSBs) and high fat foods (HFFs) and body weight in Australian children aged from 4 to 10 years.	*Design:* Longitudinal (2004, 2006, 2008, 2010)<br><br>*Data source:* Longitudinal Study of Australian Children<br><br>*Sample size:* 4,164<br><br>*Age:* 4.8 y at baseline<br><br>*Gender:* 49% female	SSB was determined by two parent reported survey questions including the frequency of consumption of fruit juice, soft drink or cordial (not diet) in the 24 hour prior to the survey.	Height and weight were objectively measured. BMIz score was derived from WHO Growth Standards.	BMI z-scores were associated with daily consumption of SSB and maternal BMI independent of BMI z-scores at wave 1 (baseline); with each additional occurrence of SSB and HFF consumption intake per day, BMI z-score increased by 0.015 U ($P < 0.01$) and 0.014 U ($P < 0.001$), respectively. With each additional maternal BMI unit, BMI z-score increased by 0.032 ($P < 0.001$).	SSB consumption did not change over the six year period. A positive association between frequency of consumption of SSBs and BMI z-scores with higher consumption was associated with a greater proportion of BMI-z scores. Effects were significant over and above the strong effect of maternal BMI.
Pase, M. P., et al. (2015).	*Design:* cross-sectional	Food frequency questionnaire. A single	Brachial blood pressure was measured three times after	Those who consumed fruit juice daily, versus rarely or	Daily use of fruit juice may increase central blood

Aim: To investigate the association of fruit juice consumption with blood pressure in community dwelling adults.	*Data source:* baseline data from fish oil multivitamin study.<br><br>*Sample size:* 160<br><br>*Age:* 59y (6)<br><br>*Gender:* 49% female	item asked how often they consumed fruit juice and categorised into rare use (combing scores from never use to no more than 3 times a month), occasional use (combining scores from once per week to 5-6 times per week), and daily use (once per day or more).	participants had been rested in a seated position for five mins. Central blood pressure was measured using applanation tonometry of the radial artery.	occasionally, had significantly higher central systolic blood pressure (F(2, 134) = 6.09, p<0.01), central pulse pressure (F(2,134) = 4.16, p<0.05), central augmentation pressure (F(2, 134) = 3.29, P<0.05) and central augmentation index (F(2,134) = 3.29, P<0.05) as well as lower pulse pressure amplification (F(2, 134) = 4.36, p<0.05).	pressure. Important because of common belief that fruit juice is healthy. Although juice may contain essential vitamins they commonly contain high amounts of sugar with negligible amounts of fibre. Juice might therefore be contributing to excessive sugar intake.
Pollard, C. M., et al. (2016).<br><br>Aim: To explore factors associated with SSB consumption in Australia.	*Design:* cross-sectional<br><br>*Data source:* WA Department of Health's Nutrition Monitor Survey Series and South Australian Monitoring and Surveillance System<br><br>*Sample size:* 13,596<br><br>*Age:* 18-65 years<br><br>*Gender:* 49.8%, 51.1% female	WA: 'In total, how many cans, bottles, glasses or cups of regular soft drink (not diet), energy drinks, sports drinks, flavoured mineral water or vitamin water, did you drink yesterday?' 'How much diet soft drink did you drink yesterday?' SA: 'on average, how much soft drink do you usually have per day?'	Self-reported height and weight from which BMI and weight status was derived, based on WHO.	Obese participants were more likely to consume SSB than healthy weight participants<br>(SA: OR=1.77; 95%CI 1.56-2.02; WA: OR=1.53; 1.05-2.24). SA obese participants consumed more<br>SSB per day (152.0 mL; 140.7-163.5) than healthy weight (80.1 mL; 73.2-88.2; p<0.001) and<br>overweight participants (106.9 mL; 99.0, 114.8; p<0.001).	Obesity was associated with SSB intake. Unclear what confounding variables were included in analysis.
Sanigorski, A. M., et al. (2007).<br><br>Aim: To examine the pattern of intake of key foods and beverages of children aged 4-12 years and the association with weight status.	*Design:* cross-sectional<br><br>*Data source:* participants were recruited from a regionally representative sample of eight kindergartens and	Computer assisted telephone interview used to assess children's usual and recent intake of soft drink and fruit juice/drink, and cordials (including energy containing flavoured	Objectively measured height and weight, BMI and weight status derived from IOTF.	Children who consumed >2–3, >3–4 and >4 servings of fruit juice/drinks 'yesterday' were, respectively, 1.7 (95% confidence interval (CI) 1.2–2.2), 1.7 (95% CI 1.2–2.5) and 2.1 (95% CI 1.5–2.9) times more likely to be overweight/obese compared with those who	Intake of sweetened beverages was associated with overweight and obesity in this population, however did not control of other dietary intake, but could have.

	18 primary schools in the Barwon South-Western Region of Victoria.<br><br>*Sample size:* 1944<br><br>*Age:* 8.3y (2.2)<br><br>*Gender:* 52.1% female	mineral water and sports drink).		had no servings of fruit juice/drink ‘yesterday’, adjusted for age, gender and socio-economic status (SES). Further, children who had $3 servings of soft drink ‘yesterday’ were 2.2 (95% CI 1.3–3.9) times more likely to be overweight/obese compared with those who had no servings of soft drink ‘yesterday’, adjusted for age, gender and SES. In addition, children who ‘usually’ drank fruit juice/drinks twice or more per day were 1.7 (95% CI 1.2–2.4) times more likely to be overweight/obese compared with those who drank these beverages once or less per week, adjusted for age, gender and SES.	
Sluyter, J. D., et al. (2013).<br><br>Aim: To clarify the relationship between lifestyle risk factors for obesity and adiposity.	*Design:* cross-sectional<br><br>*Data source:* Australian communities involved in the Obesity Prevention in Communities study.<br><br>*Sample size:* 1673<br><br>*Age:* 14.6y (1.4)<br><br>*Gender:* 39.9% female	Self-reported questionnaires ‘In the last 5 school days (including time spent at home) on how many days did you have regular (non-diet) soft drinks?’ and ‘On the last school day, how many glasses or cans of soft drinks did you have?’	Objectively measured height and weight, and categorised with WHO growth standards.	Two or more cans of soft drink per day was associated to increased BMI (b=0.46, SE=0.23, p<0.05), and increment in BMIz score (b=0.15, SE=0.07, p<0.05).	Intake of sweetened beverages (two or more cans of soft drink per day) was associated to increase BMI.

Thurber, A.K., et al. (2017)<br><br>Aim: To quantify BMI trajectories among Indigenous Australian children and identify factors associated with development of overweight/obesity.	*Design:* longitudinal<br><br>*Data source:* Australian Government Department of Social Services.<br><br>*Sample size:* 887<br><br>*Age:* 3-9 years<br><br>*Gender: 50% females*	Self-administered surveys to assess SSB consumption frequency.	Objectively measured height and weight and categorised with WHO growth standards. BMI was treated as a continuous variable.	Lower annual BMI increase for children who had fewer SSBs consumption (P=0.05, including fruit juices.)	Reducing SSB consumption from an early age can have positive impact on children's BMI trajectories.
Trapp, G. S. A., et al. (2014).<br><br>Aim: To determine the incidence of energy drink consumption and factors associated (i.e. socio-demographic status, alcohol, cigarette and illicit drug use)<br>in a young Australian adults sample.	*Design:* Cross-sectional<br><br>*Data source:* Western Australian Pregnancy Cohort (Raine)<br><br>*Sample size:* 1234<br><br>*Age:* mean age 20 ± 0.5 years<br><br>*Gender:* 53% female	Self-administered questionnaires to assess energy drink consumption patterns and frequency.	Objectively measured height and weight, BMI was treated as a continuous variable.	BMI was not significant different between energy drink users and non-users.	This study showed limited evidence of an association between energy drink users and non-users
Wheaton, N., et al. (2015).<br><br>Aim: To examine weight status<br>tracking throughout early to middle childhood and determine the<br>key influencing factors in Australian children.	*Design:* longitudinal<br><br>*Data source:* Australian children<br><br>*Sample size:* 4169<br><br>*Age:* 4-10 year-olds	SSB intake was measured using two parent-reported<br>survey questions, indicating the consumption frequency of: (1) fruit juice; (2) soft drink or cordial (not diet).	Height and weight were measured by trained research staff.<br>Abdominal obesity was classified with WHO cut-offs.	Higher consumption patterns of SSBs were linked to overweight and obesity across the time period (RRR=1.13, SE=0.06, p=0.01).	SSB were an important predictor of childhood weight status.<br>Consumption of SSBs increased over time and association to overweight/obesity was significant across the six years after controlling for covariates.

	Gender: 49% female				
Zheng, M., *et al.* (2015).<br><br>Aim: To evaluate the effects of liquid versus solid energy intake and different beverage types on changes in childhood adiposity.	*Design:* longitudinal<br>*Data source:* Childhood Asthma Prevention Study.<br>*Sample size:* 158<br>*Age:* 8- 15 year-old.<br>*Gender:* not reported	Three 24-h dietary (including beverages) recalls. ‘	Height and weight were measured by trained research staff. Overweight/obesity was defined using International Obesity Task Force cut-offs for BMI.<br>Percentage body fat was measured.	SSB consumption was associated to an increase in BMIz (b=0.10, SE=0.03, p=0.003) and body fatness (b=1.04, SE=0.32, p=0.001) from 8 to 11.5 years.	SSB consumption was associated to increased BMIz score and body fat percentage from 8 to 11.5 years after controlling for a wide range of covariates including total energy intake.

Overweight OR obes* OR adiposity OR waist circumference* OR waist-hip ratio OR body mass index OR BMI OR blood pressure OR hypertens* OR glucose intolerance OR blood glucose OR insulin OR cholesterol OR lipoprotein OR triglycerides OR lipid metabolism OR cardio metabolic OR metabolic syndrome OR cardiovascular disease* OR type 2 diabetes OR diabetes OR T2DM OR diabetes mellitus

And: carbonated beverage* OR sugar sweetened beverage* OR soft drink* OR sugar drink* OR non-diet drink* OR soda* OR soda pop OR fizzy drink* OR refreshment OR cola OR coke OR pepsi OR coca cola OR lemonade OR diet soft drink* OR diet soda OR diet cola OR diet coke OR juice OR fruit juice OR fruit drink OR orange juice OR apple juice OR energy drink OR sport drink OR gatorade OR powerade OR red bull OR dietary sucrose OR sweetening agents

And: Australia* OR Oceania OR Indigenous* OR Aboriginal* OR Torres Strait Island*

Limiters: January 1990 –August 2017

788 articles found

All databases accessed through EBSCOhost

Supplementary Figure 1. Search terms for EBSCOhost
